# Severe Hemolysis After Moderate-Dose Pulsed Field Application (48 Pulses) for Atrial Fibrillation/Flutter: A Cautionary Tale

**DOI:** 10.1016/j.jaccas.2025.105136

**Published:** 2025-09-10

**Authors:** Alireza Ghajar, Ekin C. Uzunoglu, Jan Lopes, Fabrizio Assis, Rajasekhar Nekkanti, Sorin S. Popescu, Roland Tilz, Ghanshyam Shantha, John N. Catanzaro

**Affiliations:** a Department of Cardiovascular Sciences, East Carolina University, Greenville, North Carolina, USA; b Department of Rhythmology, University Heart Center Lübeck, University Hospital Schleswig-Holstein, Kiel, Germany

**Keywords:** acute kidney injury, atrial fibrillation, atrial flutter, catheter ablation, hemolysis, pulsed field ablation

## Abstract

**Background:**

Pulsed field ablation (PFA) has gained popularity for its cardiac selectivity; however, its hemolysis safety profiles are not well understood.

**Case Summary:**

A 69-year-old, African-American man underwent atrial fibrillation/flutter ablation with moderate-dose PFA (48 pulses) and developed significant near-dialysis oliguric acute kidney injury (AKI), requiring a prolonged hospital stay.

**Discussion:**

Previous studies have noted an increased risk of hemolysis and AKI with higher PFA lesions, with 70 to 100 pulses being the historical thresholds. This case highlights that hemolysis and AKI could occur with much lower doses than previously considered safe. Universal preventive measures with empirical hydration and extended observation periods post-ablation (overnight stay) could be considered. Genetic factors that could possibly put African Americans at increased risk for hemolysis need to be explored.

**Take-Home Messages:**

Hemolysis can occur with any number of pulses with PFA. Pre-existing chronic kidney disease could be a risk factor for developing AKI.

## Visual Summary

**Figure:**
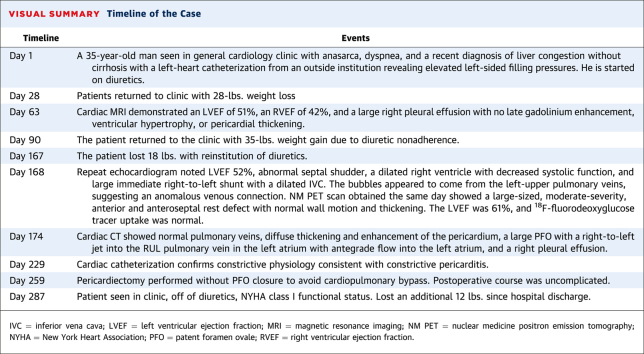

**Visual Summary:**
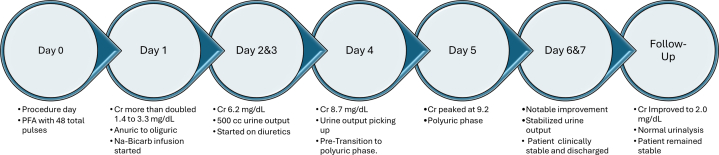
Clinical Timeline: Presentation, Labs, Management, and Follow-Up Cr = creatinine; Na-Bicarb = sodium bicarbonate infusion; PFA = pulsed field ablation.

## History of Presentation

**Figure 1:**
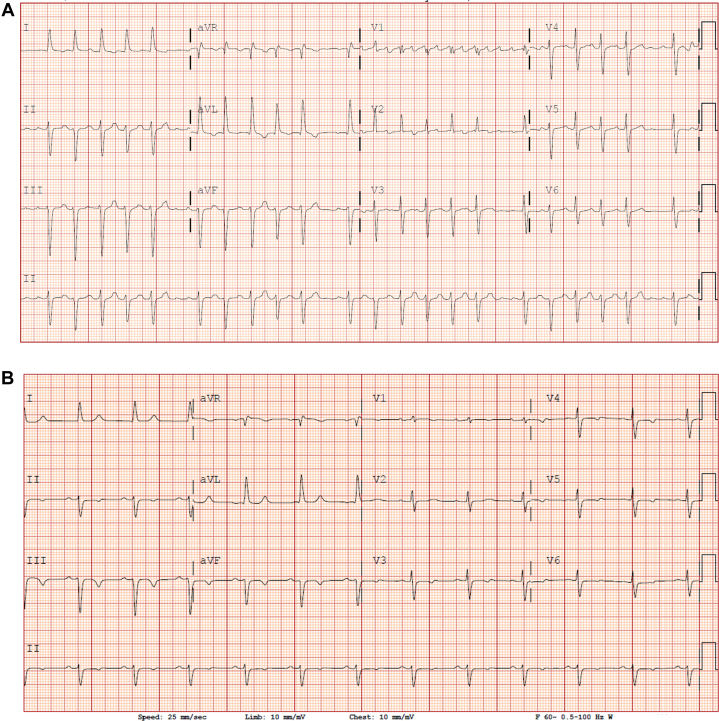
Electrocardiogram on Presentation and Post-Ablation (A) Initial electrocardiogram. (B) Electrocardiogram post-ablation procedure.

A 69-year-old African-American man presented with several days of palpitations and shortness of breath. Vital signs and physical examination were significant for elevated blood pressure (146/98 mm Hg) and tachycardia with a heart rate of approximately 150 to 160 beats/min (Figure 1A). The patient denied any chest pain, dizziness, or syncope. Baseline laboratory tests were significant for creatinine level of 1.4 mg/dL and glomerular filtration rate of 53 mL/min/1.73 m^2^. Troponin levels were within normal limits.

**Take-Home Messages**

• Hemolysis can occur with any number of pulses with PFA.
• Pre-existing chronic kidney disease could be a risk factor for developing AKI.

**Figure 2:**
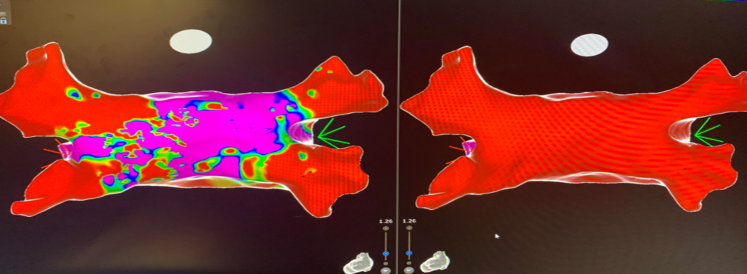
Left Atrial Mapping Pre– and Post–Pulsed Field Ablation

General anesthesia with paralytics was used. Right atrial and left atrial activation and three-dimensional mapping were performed. Intracardiac mapping and electrocardiograms revealed atrial flutter originating from the left atrium. Medium-dose pulsed field ablation (PFA) was delivered with 32 pulses (each pulse 2.0 kV) using FARAPULSE (Boston Scientific), and all 4 pulmonary veins were isolated with first pass. The entrance and exit blocks were confirmed. The posterior wall was isolated using linear ablation lines (roof and floor lines), each with 8 PFA pulses (8 × 2 = 16 pulses, each 2.0 kV) (Figure 2). A total of 48 PFA pulses were used. Fluoroscopy, intracardiac echocardiography, and intracardiac electrograms were used to assess optimal tissue contact before each energy delivery. No intraoperative hypotension occurred. Venous access to closure time was 75 minutes. Heparin was administered with activated clotting time >300 seconds during the procedure. Post-procedure electrocardiogram showed sinus rhythm (Figure 1B).

## Postoperative Day 1

The patient developed nausea, acute kidney injury (AKI), and no urine output. A bladder scan showed only 30 mL of urine. Hemoglobin dropped 4 mg/dL, from 15 to 12 mg/dL), and creatinine more than doubled, from 1.4 to 3.3 mg/dL. The patient further developed new-onset blurry vision.

## Past Medical History

Past medical history included symptomatic persistent atrial fibrillation, type 2 diabetes mellitus, hypertension, chronic kidney disease (baseline creatinine 1.4 mg/dL, glomerular filtration rate 53 mL/min/1.73 m^2^), and obstructive sleep apnea.

## Differential Diagnosis

Hemolysis and pigment-induced nephropathy were the leading differential diagnoses, supported by a significant decline in hemoglobin, oliguric tea-colored urine, and elevated lactate dehydrogenase. Other differential diagnoses included ischemic acute tubular necrosis from hypotension or transient renal hypoperfusion (less likely given that blood pressure remained stable during the procedure).

## Investigations

Laboratory tests revealed drop in hemoglobin from 15 to 12 mg/dL, doubling in creatinine from 1.4 to 3.3 mg/dL, increase in bilirubin (total 3.6 mg/dL) and lactate dehydrogenase (879 U/L), and decrease in haptoglobin (<8 mg/dL). Peripheral smear showed schistocytes. Urinalysis revealed 24 red blood cells (RBCs)/high-power field, 114 white blood cells/high-power field, +3 hemoglobin, +2 protein, and urine sodium of 88 with normal complements. Hyperkalemia (serum potassium up to 5.6) necessitated medical management to promote intracellular potassium shift. Renal ultrasound demonstrated no significant findings in the kidneys. A head computed tomography scan showed no acute abnormalities. The patient's blurry vision resolved during hospital admission. Echocardiography demonstrated a normal-sized left ventricle with preserved wall thickness and function, an ejection fraction of 50% to 55%, and a moderately dilated left atrium.

## Management (Medical/Interventions)

Following the procedure, the patient received intravenous fluid resuscitation for a few hours; however, it was complicated by dyspnea and clinical signs of volume overload. Consequently, intravenous fluids were discontinued, diuretic therapy was initiated, and a sodium bicarbonate infusion was started and continued for the next few days.

Bladder scans were routinely performed and did not show any urinary retention. By day 5, creatinine peaked at 9.2 mg/dL and began to improve, marking the transition to a polyuric phase, during which diuretic therapy was no longer required. The downtrend in creatinine continued on days 6 and 7, and the patient was discharged on day 7.

## Outcome and Follow-Up

**Figure 3:**
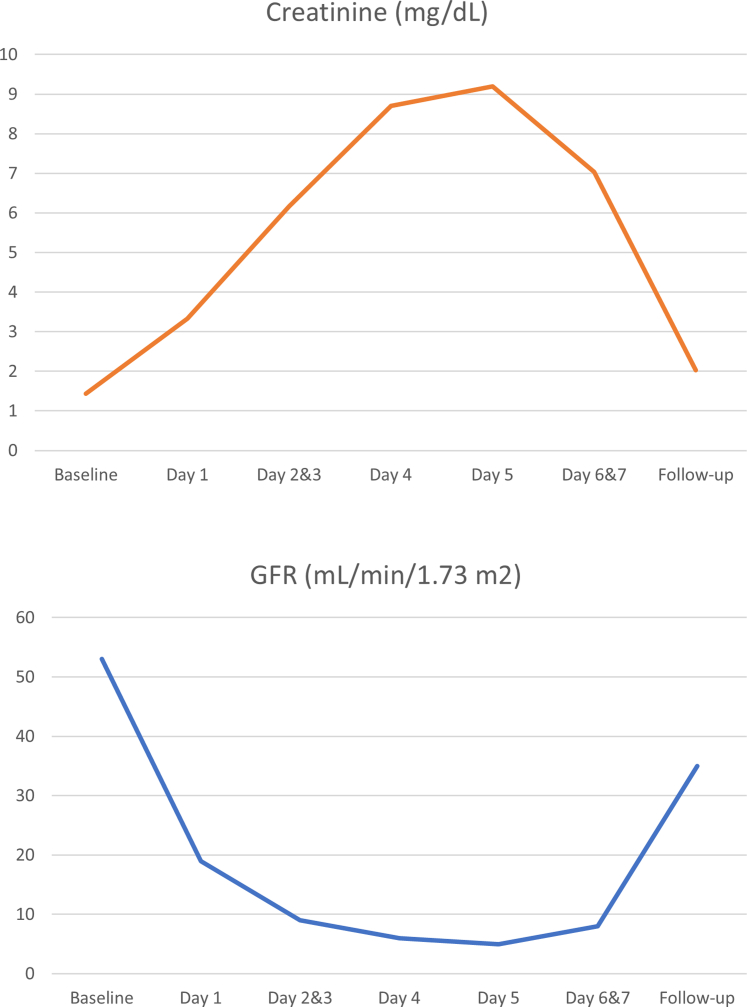
Laboratory Trends of Serum Creatinine and Estimated GFR From Baseline to Follow-Up GFR = glomerular filtration rate.

Outpatient follow-up examinations showed improved creatinine levels of 2.0 mg/dL and improved urinalysis results (urine white blood cells: none; RBCs: 0-2) 8 weeks post-procedure (Figure 3). The patient tested negative for glucose-6-phosphate dehydrogenase and sickle cell traits.

Institutional review board approval was not required for this case report in accordance with institutional policies.

## Discussion

We present a case of severe near-dialysis oliguric AKI that occurred post–moderate-dose PFA catheter ablation. PFA has gained popularity over thermal ablation for its cardiac selectivity and safety profile; however, its hemolysis safety profiles are not well understood.^1^^,^^2^ In MANIFEST-17K, a multinational survey of PFA procedures including more than 17,000 patients treated with FARAPULSE, reported zero thermal complications.^1^ However, the study showed that hemolysis-related renal failure requiring hemodialysis occurred in 5 patients in this trial; all patients recovered kidney functions.^1^

Whereas RBCs are generally resistant to mechanical stress, they are particularly susceptible to electroporation, which can cause cell damage and lead to the release of hemoglobin (hemolysis) depending on the strength of the applied electrical field.^2^ Hemolysis caused by electroporation occurs secondary to high-voltage pulses causing hundreds of pores in erythrocyte membranes, enabling uncontrolled ion and osmolyte flux. This leads to cell swelling to a critical volume and instant membrane disruption through cytoplasmic jet expulsion.^3^ In vivo and ex vivo data validate that PFA electroporation may result in extreme intravascular hemolysis.^4^, ^5^, ^6^ Hemolysis releases free hemoglobin, which, under usual haptoglobin scavenging, may be benign; however, under excessive hemolysis, this mechanism is overwhelmed, resulting in methemoglobin, oxidative stress, and measurable hemoglobinuria.^7^ In patients with borderline renal function or increased RBC fragility (eg, glucose-6-phosphate dehydrogenase deficiency, sickle cell trait [more prevalent in African Americans]), this process is clinically relevant. Released free hemoglobin and heme potentially serve as nephrotoxins, causing oxidative tubular injury, causing obstruction via pigment casts, and triggering inflammatory cascades, resulting in AKI.^4^

According to initial reports of patients with AKI who required dialysis in the MANIFEST-17K trial, the number of applications those patients received was 143 ± 27. Prior studies indicate that the determining factor for the severity of hemolysis is the number of applications.^6^^,^^8^ Venier et al^6^ reported 2 AKI cases after PFA applications of 174 and 126; reporting up to 70 PFA applications seemed reasonable to avoid hemolysis complications. The same results were reported in the study by Osmancik et al,^5^ who found that the likelihood of significant renal injury is low with 70 PFA lesions. Later, Popa et al^2^ reported that PFA is associated with substantial intravascular hemolysis, with 54 or more PFA deliveries linked to increased severity, particularly in patients with a baseline glomerular filtration rate <50 mL/min, raising concerns about potential nephrotoxic effects owing to creatinine changes, AKI, and hemoglobinuria. Planned hydration post-ablation could decrease the risk of renal insult in patients undergoing PFA^8^; however, this might need to be investigated in more extensive trials.

On the other hand, the efficiency of PFA catheter designs by computer modeling suggests that balloon catheters could be more efficacious and safer than pentaspline PFA catheters, which need more investigation.^9^

## Conclusions

PFA is a developing technology in the treatment of atrial fibrillation with the potential to reduce collateral damage and procedural time compared with traditional thermal ablation methods. This case highlights that hemolysis and AKI could occur in much lower doses than previously considered safe. Universal preventive measures with empirical hydration or extended observation periods post-ablation (overnight stay) could be considered.

## Funding Support and Author Disclosures

Dr Tilz has been a consultant for Abbott, Boston Scientific, Biotronik, and Biosense Webster and has received speaker honoraria payment support for meetings or travel from Biosense Webster, Medtronic, Boston Scientific, and Abbott Medical, and research support from Abbott Medical. Dr Catanzaro has received research funding from Aziyo Biologics, honoraria from the University of Florida Research Foundation, and has received royalties for licensed intellectual property. All other authors have reported that they have no relationships relevant to the contents of this paper to disclose.
